# Neuroanatomy from Mesoscopic to Nanoscopic Scales: An Improved Method for the Observation of Semithin Sections by High-Resolution Scanning Electron Microscopy

**DOI:** 10.3389/fnana.2018.00014

**Published:** 2018-02-27

**Authors:** José-Rodrigo Rodríguez, Marta Turégano-López, Javier DeFelipe, Angel Merchán-Pérez

**Affiliations:** ^1^Laboratorio Cajal de Circuitos Corticales, Centro de Tecnología Biomédica, Universidad Politécnica de Madrid (UPM), Madrid, Spain; ^2^Instituto Cajal, Consejo Superior de Investigaciones Científicas (CSIC), Madrid, Spain; ^3^Departamento de Arquitectura y Tecnología de Sistemas Informáticos, Universidad Politécnica de Madrid (UPM), Madrid, Spain

**Keywords:** brain mapping, electron microscopy, scanning electron microscopy (SEM), FIB-SEM, light microscopy, semithin sections, neuroanatomy

## Abstract

Semithin sections are commonly used to examine large areas of tissue with an optical microscope, in order to locate and trim the regions that will later be studied with the electron microscope. Ideally, the observation of semithin sections would be from mesoscopic to nanoscopic scales directly, instead of using light microscopy and then electron microscopy (EM). Here we propose a method that makes it possible to obtain high-resolution scanning EM images of large areas of the brain in the millimeter to nanometer range. Since our method is compatible with light microscopy, it is also feasible to generate hybrid light and electron microscopic maps. Additionally, the same tissue blocks that have been used to obtain semithin sections can later be used, if necessary, for transmission EM, or for focused ion beam milling and scanning electron microscopy (FIB-SEM).

## Introduction

During preparation of tissue for electron microscopy (EM), semithin sections (usually 0.5–2 μm thick) are commonly obtained for observation under the light microscope. These semithin sections are used to examine large regions of the tissue with an optical microscope, in order to locate and trim the areas that will later be studied with EM. Ideally, the observation and analysis of semithin sections would be from mesoscopic to nanoscopic scales directly, instead of using light microscopy and then EM. The problem is the limitation imposed by the low resolution of optical microscopy. To overcome this limitation, several methods have been developed for the observation of semithin sections under a scanning electron microscope (SEM; Pasquinelli et al., 1985; Koga et al., 2015; Rizzo et al., 2016). Although similar techniques have also been described for ultrathin sections (29–35 nm thick) (Schalek et al., 2011; Horstmann et al., 2012), we have focused on methods using semithin sections since they allow much larger sections to be obtained, making it possible to examine wider areas of tissue. Moreover, semithin sections can be studied and imaged with a light microscope before being observed under the SEM, so they are suitable for correlative light and EM.

Our present work is based on previous developments described recently (Koga et al., 2015; Rizzo et al., 2016) and addresses two important issues that we found when using these methods. The issues—which occur at two critical steps of the general procedure—are as follows: first, the sections must be collected on supporting media that allow further staining and imaging. Silicon wafers as a collecting support have been described as being superior to glass cover slips for SEM imaging, but they are not suitable for correlative light microscopy. On the other hand, glass cover slips are less stable and pose more charging issues than silicon wafers (Rizzo et al., 2016), and they need to be manually cut with a diamond pen before mounting on the SEM specimen stubs (Koga et al., 2015). Second, in order to facilitate charge dissipation and to prevent charge build-up artifacts, sputter coating of the specimen with carbon or heavy metals is commonly used. In our experience, however, the thickness of this conductive coating must be carefully adjusted, since a layer that is too thin will not prevent charge artifacts, and a layer that is too thick will prevent high-resolution imaging of the underlying tissue section with the SEM (Merchán-Pérez et al., 2009).

To overcome the two issues described above, here we propose to collect the semithin sections on a flat epoxy resin sheet that has been previously sputter-coated with heavy metals. Semithin sections collected on this resin sheet can be stained with toluidine blue and examined and photographed under an optical microscope. The resin sheet supporting the semithin section is easy to manipulate, crop and glue to the SEM specimen stubs. Moreover, the conductive coating is applied *before* collecting the semithin sections, so it lies *below* the specimen and, as a result, it does not obscure high-resolution SEM imaging. This modification represents a significant, practical improvement on the original methods and deals effectively with the issues encountered. Finally, once the desired semithin sections have been obtained, the remaining block can—if necessary—also be used for conventional transmission EM or for combined focused ion beam milling and scanning electron microscopy (FIB-SEM; Langford, 2006; Knott et al., 2008; Merchán-Pérez et al., 2009).

## Methods

### Tissue Preparation

Four adult (8 weeks), male mice (C57BL/6) were used in this study. All animals were handled in accordance with the guidelines for animal research set out in the European Community Directive 2010/63/EU, and all procedures were approved by the local ethics committee of the Spanish National Research Council (CSIC). The animals were administered a lethal intraperitoneal injection of pentobarbital (40 mg/kg) and were intracardially perfused with 4% paraformaldehyde in 0.12 M phosphate buffer, pH 7.4 (PB). After perfusion, the brains were extracted from the skull and left overnight at 4°C in the same fixative. The sections (150 μm thick) were obtained with a vibratome, collected in PB and washed in 0.1 M cacodylate buffer (CB), pH 7.4, 2 × 10 min. These sections were subsequently postfixed in a microwave oven (Pelco Biowave Pro) in 2% paraformaldehyde and 2.5% glutaraldehyde in CB containing 0.003 M CaCl_2_. They were then osmicated in CB with 1% OsO_4_, 0.1% potassium ferricyanide and 0.003 M CaCl_2_ for 1 h, and washed three times in CB. A second postfixation was then performed with the same concentrations of OsO_4_ and CaCl_2_ for an additional hour. After three rinses in CB, the vibratome sections were dehydrated with a graded series of ethanol and en-bloc stained with 1% uranyl acetate in 50%, 70% and 90% ethanol. The sections were finally cleared with acetone and flat-embedded in Araldite (DeFelipe and Fairén, 1993).

### Preparation of Glass Microscope Slides

**Figure 1:**
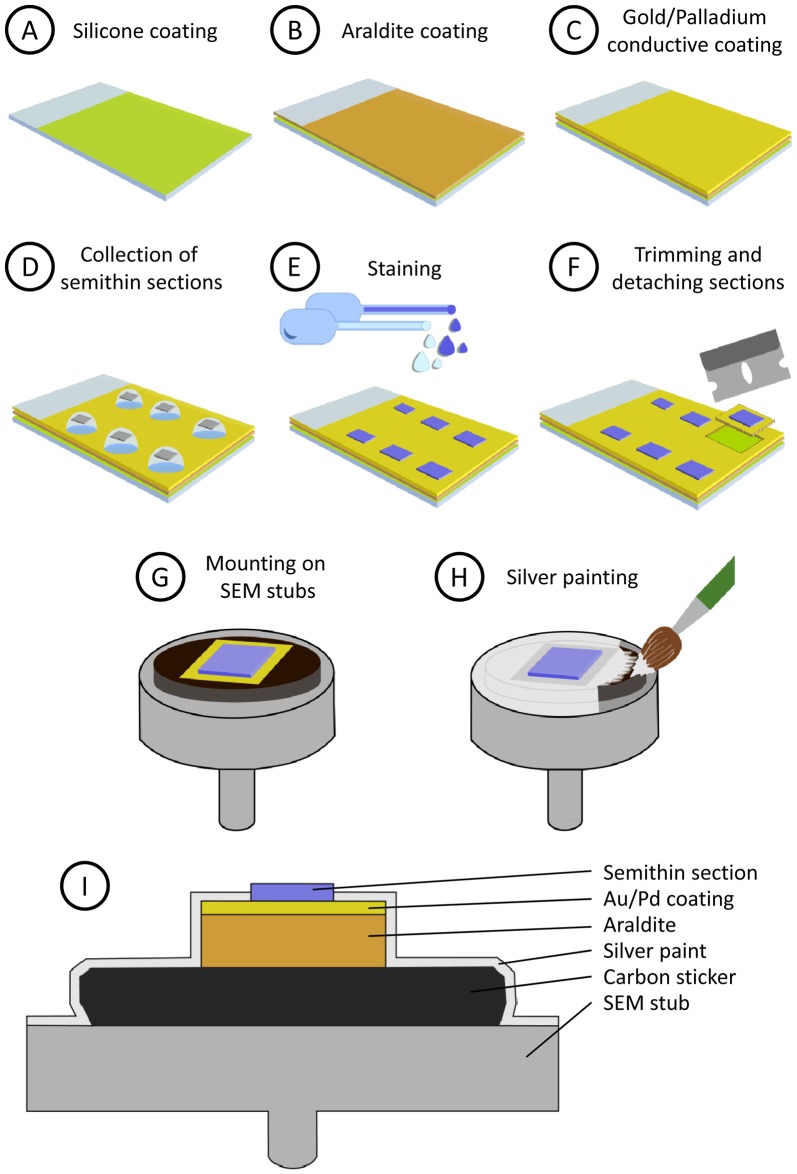
Step-by-step preparation and mounting of semithin sections (see “Methods” section). **(A)** Microscope glass slides are covered with silicone. **(B)** A layer of Araldite is poured and polymerized on the siliconized glass slide. **(C)** The glass slide is then sputter-coated with gold/palladium to make it conductive. **(D)** Semithin sections are collected on water drops and dried on a hot plate. **(E)** Semithin sections are stained for light and electron microscopy (EM). **(F)** Selected sections are trimmed and detached from the slide. Note that the silicon layer prevents the Araldite from firmly adhering to the glass, so the square of Araldite supporting the section can be detached easily. **(G)** Mounting of the semithin section on an aluminum scanning electron microscopy (SEM) stub. **(H)** Conductive silver painting to avoid charge build-up. **(I)** Summarized depiction of the semithin section once mounted on the SEM stub (not to scale).

Glass microscope slides are first covered with silicone, then with a layer of araldite and finally they are sputter-coated with heavy metals. Silicone prevents Araldite from adhering to the glass surface, so the resin layer can be easily trimmed and detached from the glass slide when necessary. The heavy metal coating helps to prevent charge build-up artifacts when the sample is observed with the SEM. The step-by-step procedure is as follows (see also Figure 1):

1. Preparation of silicone-covered microscope slides (Figure 1A). Microscope glass slides are immersed in silicone (Serva, 35130) for a few seconds and then placed vertically in a slide holder (Ted Pella, 21078-1) to let them drain. When they are still wet, they are placed in an oven at 100°C for 1–2 h. Silicone-covered glass slides can be stored for months in dust-free slide boxes.
2. Preparation of a flat Araldite bed on silicone-covered microscope slides (Figure 1B). To cover siliconized slides with Araldite, acetate film pieces (Aclar, 10501, Ted Pella Inc., Redding, CA, USA) are trimmed to a size that is slightly smaller than the glass slides. Araldite is freshly prepared and degassed in a vacuum chamber. A few drops of resin are placed on the acetate sheets and they are turned upside down on the glass slides. The acetate sheets are then gently pressed to spread the resin and to eliminate air bubbles, so the araldite layer below the acetate sheet is flat and uniform. Then, polymerization of Araldite is carried out in an oven at 60°C for 48 h. After polymerization, the acetate film is removed and Araldite-covered slides are stored until needed.
3. Conductive coating of Araldite (Figure 1C). Araldite-covered slides are coated with gold-palladium for 120 s in a sputter coater (Quorum Emitech SC7620). The slides are now ready for the semithin section collection step. Note that the conductive gold-palladium coating will lie below the sections once they are collected.

### Collection and Staining of Semithin Sections

4. Semithin sectioning. Semithin sections (0.5 microns thick) are obtained with an ultramicrotome (Leica MZ6) and a diamond knife (Diatome Histo, 6 mm). They are collected on water drops on the previously prepared slides (Figure 1D). The slides are then placed on a hot plate at 60°C until the water has dried and the sections have adhered to the supporting resin. No gluing agent is necessary and, indeed, it must be avoided so that the section lies directly on the gold-palladium coating.
5. Staining (Figure 1E). For observation under the light microscope, the semithin sections are stained with 0.125% toluidine blue in 0.25% borax, examined under the light microscope and photographed. The sections that are selected for further study with the electron microscope are then stained with a 1% aqueous solution of uranyl acetate for 10 min and a 1% aqueous solution of lead citrate for 5 min.

### Preparation of Semithin Sections for SEM

6. Trimming and detaching sections from the glass slide. The araldite around the selected semithin section is cut with a single-edged cutter blade. The square of Araldite supporting the section is then detached from the glass slide (Figure 1F). This operation is facilitated by the presence of the silicone layer, since it prevents adhesion between glass and Araldite, so the sections can be easily detached once they have been trimmed with the knife.
7. Mounting of semithin sections on SEM specimen stubs (Figure 1G). The sections are glued onto SEM stubs (Electron Microscopy Sciences, 75191) with a conductive carbon sticker (Electron Microscopy Sciences, 77825-09).
8. Silver painting. To facilitate charge dissipation, electrical continuity between the gold coated Araldite and the aluminum stub must be ensured. To achieve this, the resin around the semithin section, the conductive sticker and the upper face of the aluminum stub are covered with silver paint (Electron Microscopy Sciences, 12630). Care must be taken not to cover the semithin section itself with silver paint (Figures 1H,I). The paint is dried for at least 24 h in a vacuum desiccator, where the stubs with the sections are stored until observation under the SEM.

### SEM Observation

A field emission SEM column (Gemini II, Zeiss, Germany) was used for the present study. We used the secondary electron detector (SE2) and the in-lens back-scattered electron detector (BSE) at acceleration voltages ranging from 1.7 kV to 8 kV. Original image size was 2048 × 1536 pixels. Noise reduction was performed by either line averaging or frame integration. Total image acquisition times per photomicrograph ranged from 1 min to 3 min.

## Results and Discussion

### Methodological Considerations

Preparation of semithin sections to be studied by the present method is easy and reliable and it can be carried out with essentially the same equipment needed for conventional EM. We recommend perfusion fixation followed by vibratome sectioning and flat embedding. However, fixation by immersion can also be used, which is particularly appropriate when using human brain tissue. In the present method, perfusion fixation has been performed with paraformaldehyde alone, so alternative sections can be used for immunocytochemical methods and confocal microscopy, or for EM. Our method, however, is compatible with the use of glutaraldehyde during perfusion (for example, 2% paraformaldehyde and 2.5% glutaraldehyde). The vibratome sections containing the regions of interest are postfixed in a mixture of paraformaldehyde and glutaraldehyde, and then in osmium tetroxide, and further processed for EM. We also use a low concentration of potassium ferricyanide and an “en bloc” staining step with uranyl acetate. Although these staining steps (potassium ferricyanide and uranyl acetate) are not essential for the subsequent observation of semithin sections with the SEM, they do increase image quality if the same samples are later imaged by FIB-SEM. In fact, the present method can be used to locate the exact regions that will later be studied by FIB-SEM, as in Supplementary Videos 1–3. Semithin sections thicker than 0.5 μm can also be used. However, we recommend 0.5 μm thick sections because image quality is optimal in that case, probably due to a better charge drainage through thinner sections.

One of the main advantages of our method over similar methods is that we collect semithin sections on Araldite-covered microscope slides. In this way, the sections can be stained with toluidine blue so they can be studied with a light microscope and selected for further study under the SEM. If sections are collected directly on glass, they cannot be detached for observation with the SEM, so the glass coverslip needs to be trimmed with a diamond tool (Koga et al., 2015). In our experience, this is difficult to perform and coverslips often break unintentionally. Moreover, according to Rizzo et al. (2016), glass is not recommended since it is less stable and poses more charging issues, so they favor the use of silicon wafers. However, silicon wafers cannot be studied under the light microscope. In our case, the araldite layer supporting the semithin section is separated from the microscope glass slide by a thin layer of silicone that prevents the Araldite from firmly adhering to the glass. In this way, the square of Araldite that contains the semithin section is easy to trim and detach from the slide, so it can be glued onto the SEM stub later.

Another important difference of our method compared to similar methods, in particular those of Koga et al. (2015) and Rizzo et al. (2016), is that we apply the conductive sputter coating *before* we collect the sections, so the coating lies *under* the section and so it does not interfere with image acquisition. It is certainly possible to apply the conductive coating over the sections, but this is—in our experience—a delicate procedure since it is very difficult to control: too little coating will not avoid charge artifacts, while too much coating will obscure image details. In our method, a relatively thick Gold/Paladium coating lies directly below the sample, and this is in practice sufficient to ensure charge dissipation. In other words, the upper surface of the semithin section is directly exposed to the electron beam with no interference from carbon or heavy metal coating.

### Image Acquisition from Semithin Sections

**Figure 2:**
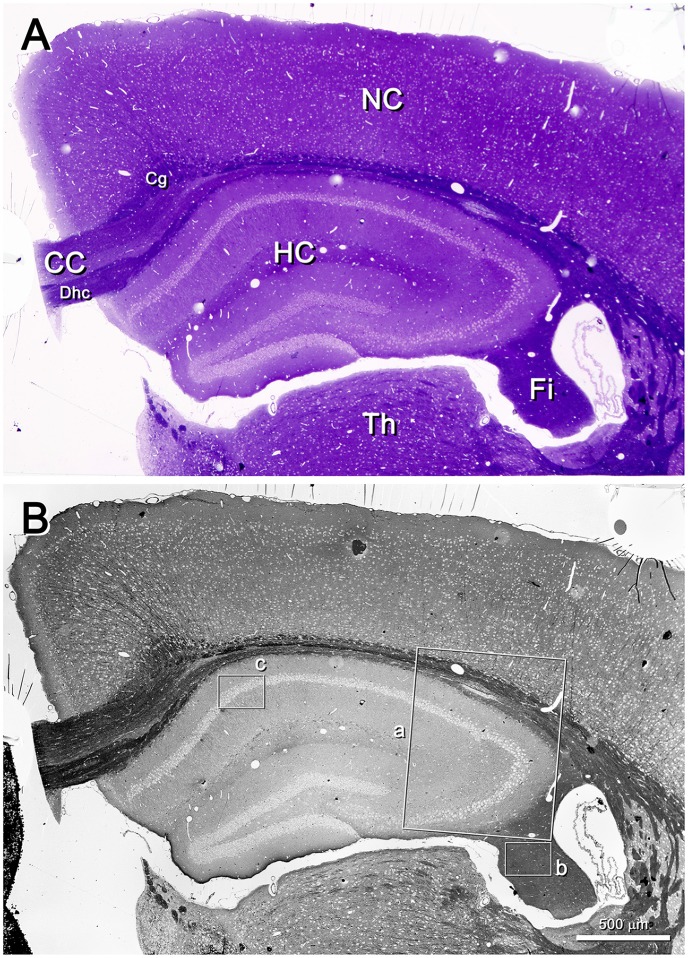
Semithin section of the mouse brain. **(A)** Toluidine blue-stained semithin section (0.5 μm thick) photographed with a light microscope. The resolution is good enough to identify gross structures such as the hippocampus (HC), the neocortex (NC) and thalamus (Th). White matter subdivisions are also visible, such as the corpus callosum (CC), dorsal hippocampal commissure (Dhc), cingulum (Cg) and fimbria (Fi). **(B)** The same semithin section imaged with a SEM. The SE2 detector has been used, with an acceleration voltage of 8.00 kV. The lighter profiles correspond to cell somata, while the darkest structures correspond to white matter. Original magnification was 30×, equivalent to a resolution of 1.86 μm/pixel. The original field of view was 3.811 × 2.858 mm; the frame was later slightly cropped horizontally. The insets a, b and c have been further imaged in Figures 3, 4, 6, respectively.

**Figure 3:**
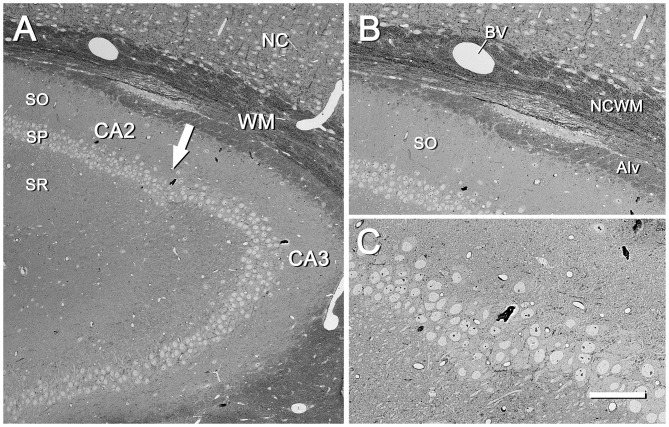
CA2/CA3 region of the mouse hippocampus. **(A)** CA2/CA3 regions corresponding to the inset “a” of Figure 2. SO, stratum oriens; SP, stratum pyramidale; SR, stratum radiatum; NC, neocortex; WM, white matter. The arrow points to the boundary between CA2 and CA3. The original image was acquired with the SE2 detector at 70× (resolution 792.2 nm/pixel), with an acceleration voltage of 8.00 kV. **(B)** Higher magnification of the same region shown in **(A)**, where differences in the architectural features of the fascicles of fibers can be seen between the neocortical white matter (NCWM) and the alveus (Alv). SO, stratum oriens; BV, blood vessel. The original image was taken with the SE2 detector at 204× (273.4 nm/pixel), accelerating voltage of 8.00 kV. **(C)** Higher magnification of the SP at the boundary between CA2 and CA3. Original image acquired with the SE2 detector at 400× (139.6 nm/pixel), accelerating voltage of 8.00 kV. Calibration bar: 140,100 and 50 μm for **(A)**, **(B)** and **(C)**respectively.

**Figure 4:**
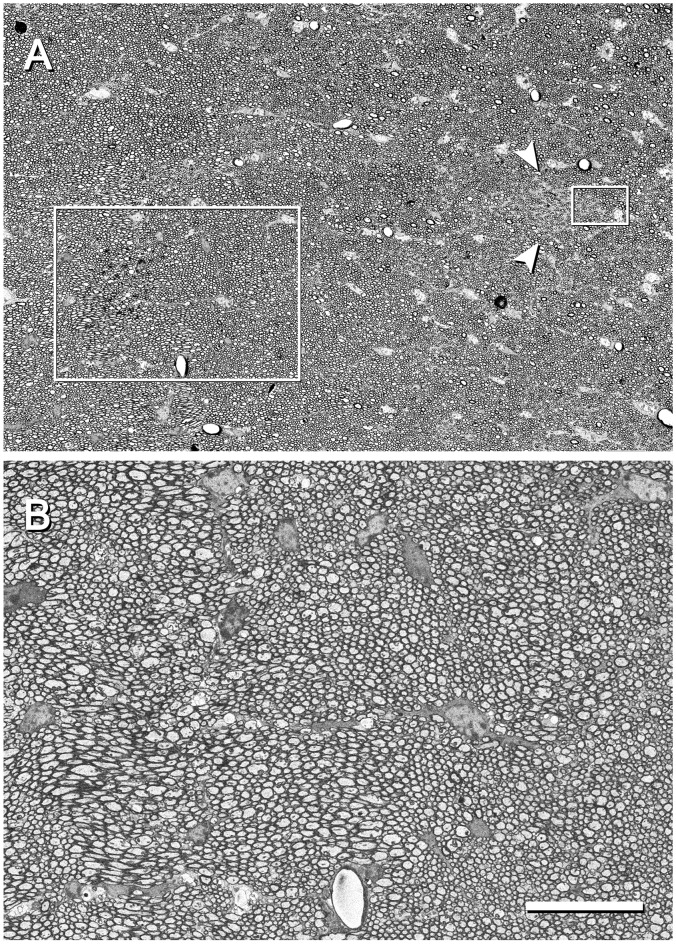
Fimbria hippocampi of the mouse. **(A)** This image was acquired from the inset “b” of Figure 2 with the SE2 detector at 400× (resolution of 139.6 nm/pixel), and an acceleration voltage of 3.0 kV. Numerous round or elliptic profiles corresponding to myelinated axons of different calibers are visible running in loosely defined bundles. Glial cells distributed throughout the whole field are also visible. Arrowheads point to a small zone that is enriched with unmyelinated fibers (see also Figure 5). **(B)** Higher magnification of the large boxed area in **(A)** illustrating bundles of fibers of different calibers and orientations. The image was acquired with the back-scattered electron detector (BSE) at 600× (resolution of 93.04 nm/pixel), with an acceleration voltage of 3.00 kV. Calibration bar: 50 μm for **(A)** and 18 μm for **(B)**. The small boxed area in **(A)** is further magnified in Figure 5.

**Figure 5:**
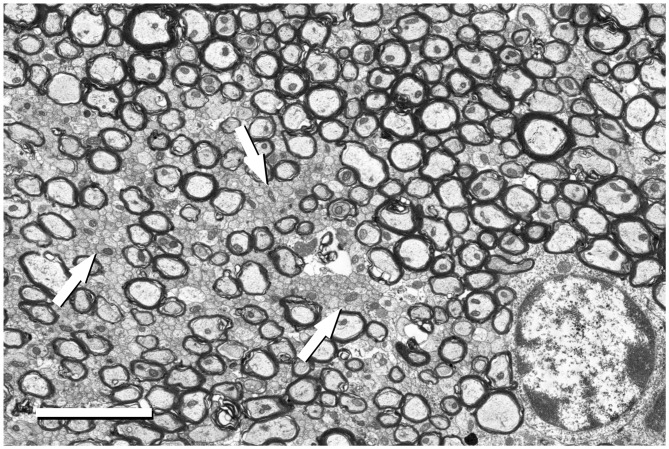
Detail of the fimbria hippocampi of the mouse. This image was acquired from the small boxed area of Figure 4A with the BSE detector at 5050× (resolution of 11.05 nm/pixel), with an acceleration voltage of 2.00 kV. Bundles of unmyelinated fibers (arrows) which are intermingled between myelinated fibers can be seen. The cell body of an oligodendrocyte is visible in the bottom right corner. Calibration bar: 4 μm.

**Figure 6:**
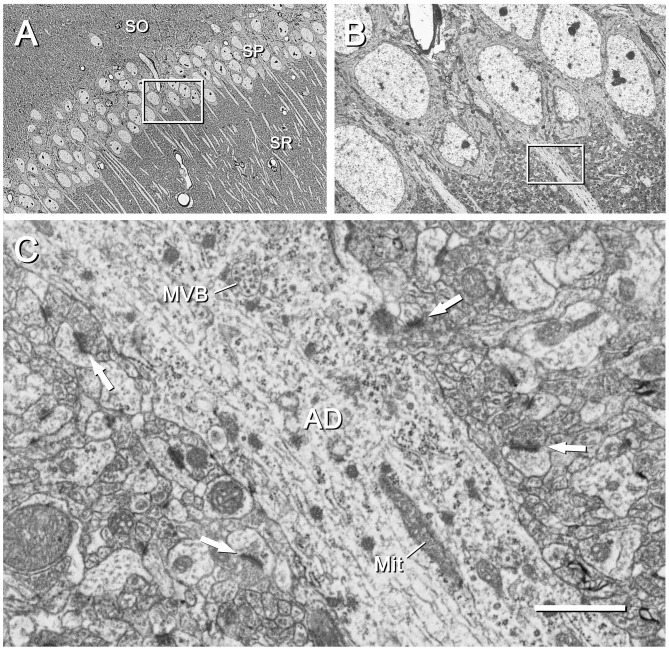
CA1 region of the mouse hippocampus. **(A)** Area of the CA1 region corresponding to the inset “c” of Figure 2. The cell bodies and apical dendrites of pyramidal cells are clearly visible. The inset has been further magnified in **(B)**. SO, stratum oriens; SP, stratum pyramidale; SR, stratum radiatum. The original image was acquired with the SE2 detector at a magnification of 439× (resolution of 127.2 nm/pixel), with an acceleration voltage of 8 kV. **(B)** Higher magnification of the stratum pyramidale shown in **(A)**. The inset on the apical dendrite has been further magnified in **(C)**. The original micrograph was taken with the SE2 detector at 2500× (resolution of 22.33 nm/pixel), with an acceleration voltage of 2.2 kV. **(C)** Apical dendrite (AD) of a pyramidal cell and the surrounding neuropil, as framed by the inset in **(B)**. Subcellular organelles such as a multivesicular body (MVB) and mitochondria (Mit) are clearly visible. Synaptic junctions in the surrounding neuropil are also visible; some of the postsynaptic densities are marked with arrows. The original image was acquired with the BSE detector at 15,000× (resolution of 3.72 nm/pixel), with an acceleration voltage of 1.8 kV. Calibration bar: 34 μm for **(A)**, 6 μm for **(B)**, 1 μm for **(C)**.

Low-power images (from 25× onwards) can be obtained with the SE2 detector, using acceleration voltages ranging from 2 kV to 8 kV. These images are very useful because they provide a panoramic view where the structures of interest can be easily identified. For example, in Figure 2 the field of view is wide enough to show the entire semithin section that had previously been photographed under the light microscope. In this example, the mouse hippocampus and surrounding structures are clearly identified in both the light and electron photomicrographs (Figures 2A,B, respectively). This particular SEM image was originally taken at 30×, which is equivalent to a resolution of 1.861 μm per pixel, giving a field of view of 3.811 × 2.858 mm. These low-power images allow the identification of gross structures such as the neocortex, the hippocampus or the corpus callosum in the same field. Thus, it is feasible to focus on particular structural aspects of these regions, such as the borders between CA2 and CA3 or the variations of the white matter architecture between the neocortex, the hippocampus (Figure 3) and fimbria (Figures 4, 5). Small structures such as neuronal somata, blood vessels and fiber bundles can also be visualized at these low powers. With our equipment, the SE2 detector can be used up to approximately 8000× with good results. However, from approximately 2000× up to 15,000×, the BSE detector gives better results than the SE2 detector (Figures 5, 6). Fine detail is maintained throughout all the scales of magnification, so—for example—subcellular structures such as synapses, mitochondria and multivesicular bodies can be identified (Figure 6). Even the two major types of cortical synapses, asymmetric and symmetric synapses (Peters et al., 1991), can be readily distinguished. Therefore, with the present method, magnifications ranging from approximately 30× to 15,000× are easily obtained with the SEM, thus bridging the gap between light and EM.

### A Method Suitable for Hybrid Light and Electron Microscopic Maps

In summary, here we have described a method that makes it possible to obtain high-resolution images of brain regions in the millimeter to nanometer range. Since these images are obtained from the same sections, in practice it is like zooming and panning at different levels of resolution through different brain regions. Since our method is also compatible with light microscopy, it is feasible to generate hybrid light and electron microscopic maps. There are hundreds of brain regions and subregions and many of them have not yet been explored at the ultrastructural level. The present method will allow relatively rapid inspection of multiple areas. Even the whole hemisphere of small brains like that of the mouse could be included in a semithin section prepared for SEM. This is important not only to further examine the normal brain at the ultrastructural level—such as, for example, the border zones between different regions and subregions—but also to examine changes after different experimental or pathological conditions. Indeed, possible ultrastructural changes under different experimental conditions are usually evaluated in only one particular region like, for example, a certain layer of the CA1 field of the hippocampus. This is because the sections prepared for EM have to be trimmed to a relatively small block, losing a large portion of the material. However, with the present method, it is possible to first evaluate at different levels of magnification and then select specific regions or subregions to be further examined using conventional transmission EM or FIB-SEM. Finally, this method can be applied to help study the human brain by conventional EM or by FIB-SEM, as it has been shown that FIB-SEM can be used to examine brain tissue obtained from autopsy (Blazquez-Llorca et al., 2013), which is the most common source of human tissue for research.

## Author Contributions

J-RR, JD and AM-P designed the research. J-RR and MT-L performed the research. J-RR, MT-L, JD and AM-P prepared the manuscript and figures.

## Conflict of Interest Statement

The authors declare that the research was conducted in the absence of any commercial or financial relationships that could be construed as a potential conflict of interest.

## Supplementary Material

Supplementary Videos 1–3 White matter of the somatosensory cortex of the mouse imaged with combined Focused Ion Beam Milling and Scanning Electron Microscocopy (FIB/SEM). Note the different orientations of the fascicles of myelinated fibers. The videos can be downloaded here: http://cajalbbp.cesvima.upm.es/storage/WhiteMatterVideos/White%20matter%201.mp4 http://cajalbbp.cesvima.upm.es/storage/WhiteMatterVideos/White%20matter%202.mp4 http://cajalbbp.cesvima.upm.es/storage/WhiteMatterVideos/White%20matter%203.mp4
